# Anti-PSMA CAR-Engineered NK-92 Cells: An Off-the-Shelf Cell Therapy for Prostate Cancer

**DOI:** 10.3390/cells9061382

**Published:** 2020-06-02

**Authors:** Isabella Monia Montagner, Alessandro Penna, Giulio Fracasso, Debora Carpanese, Anna Dalla Pietà, Vito Barbieri, Gaia Zuccolotto, Antonio Rosato

**Affiliations:** 1 Veneto Institute of Oncology IOV-IRCCS, 35128 Padua, Italy; isabellamonia.montagner@iov.veneto.it (I.M.M.); alessandro.penna.1994@gmail.com (A.P.); debora.carpanese@gmail.com (D.C.); 2 Department of Medicine, University of Verona, 37134 Verona, Italy; giulio.fracasso@univr.it; 3 Department of Surgery, Oncology and Gastroenterology, University of Padua, 35128 Padua, Italy; anna.dallapieta@gmail.com (A.D.P.); vito.barbieri@unipd.it (V.B.)

**Keywords:** cancer immunotherapy, prostate cancer, CAR, PSMA, NK-92 cell line

## Abstract

Prostate cancer (PCa) has become the most common cancer among males in Europe and the USA. Adoptive immunotherapy appears a promising strategy to control the advanced stages of the disease by specifically targeting the tumor, in particular through chimeric antigen receptor T (CAR-T) cell therapy. Despite the advancements of CAR-T technology in the treatment of hematological malignancies, solid tumors still represent a challenge. To overcome current limits, other cellular effectors than T lymphocytes are under study as possible candidates for CAR-engineered cancer immunotherapy. A novel approach involves the NK-92 cell line, which mediates strong cytotoxic responses against a variety of tumor cells but has no effect on non-malignant healthy counterparts. Here, we report a novel therapeutic approach against PCa based on engineering of NK-92 cells with a CAR recognizing the human prostate-specific membrane antigen (PSMA), which is overexpressed in prostatic neoplastic cells. More importantly, the potential utility of NK-92/CAR cells to treat PCa has not yet been explored. Upon CAR transduction, NK-92/CAR cells acquired high and specific lytic activity against PSMA-expressing prostate cancer cells in vitro, and also underwent degranulation and produced high levels of IFN-γ in response to antigen recognition. Lethal irradiation of the effectors, a safety measure requested for the clinical application of retargeted NK-92 cells, fully abrogated replication but did not impact on phenotype and short-term functionality. PSMA-specific recognition and antitumor activity were retained in vivo, as adoptive transfer of irradiated NK-92/CAR cells in prostate cancer-bearing mice restrained tumor growth and improved survival. Anti-PSMA CAR-modified NK-92 cells represent a universal, off-the-shelf, renewable, and cost-effective product endowed with relevant potentialities as a therapeutic approach for PCa immunotherapy.

## 1. Introduction

Remarkable therapeutic responses have been recently achieved with T cells expressing chimeric antigen receptors (CARs) to tumor antigens, especially in patients with lymphoid malignancies [1,2,3,4]. Although these data highlight the potentiality of immune cells to translate into powerful therapeutic agents, nonetheless successes in the hematological setting have yet to find full validation in solid tumors. Moreover, apart from a relevant association of CAR-T cell treatment with adverse events including a potentially fatal “cytokine release syndrome”, the logistics and costs of this approach pose significant challenges to make it available to a wide number of patients.

An increasing number of investigators believe that natural killer (NK) cells obtained from the peripheral blood of either the patient (autologous) or a healthy donor (allogeneic), might represent safer effectors for targeted cancer therapy than T cells. Additionally, the availability of continuously expanding NK cell lines provides a potentially unlimited source of effector cells, which can be investigated for genetic engineering [5,6,7], but also hold the potential for the development as standardized off-the-shelf therapeutics for adoptive cancer immunotherapy (ACT). Among such NK cell lines, NK-92 cells are those that have been most thoroughly investigated and have already reached the testing phase in the clinical setting [8,9,10,11]. In this scenario, CAR-engineered NK-92 cells could offer a valid and cost-effective alternative to primary CAR NK or T cells, in particular in those cases where a suitable donor is not available or the sophisticated infrastructures needed for cell isolation, expansion, and genetic modification are lacking. In this regard, the methodologies for continuous good manufacturing practice (GMP)-compliant expansion from an established master cell bank have been validated in the framework of early phase clinical trials with unmodified NK-92 cells, and can be easily adapted for large-scale production in centralized facilities [8,12]. This provides a further advantage that may be readily extended to CAR-engineered NK-92 variants. Indeed, in contrast to CAR approaches based on autologous or donor-derived primary cells, the genetic modification of NK-92 cells is not performed in a patient-individual setting under tight time constraints. Instead, a molecularly and functionally well-characterized cell product can be established that is endowed with a particular target specificity, and is continuously available independently from the time point of therapeutic application [13].

Here, we investigated the potential role of genetically CAR-modified NK-92 cells as a therapeutic approach for PCa, the most common cancer among males in Europe and the USA [14,15,16]. The specific targeting of this tumor by ACT represents a promising strategy to control the disease, as PCa expresses distinct surface tumor-associated antigens that can be exploited for immune interventions. Among these, the expression levels of the prostate-specific membrane antigen (PSMA) differentiate normal and cancerous prostatic tissues, and parallel the Gleason score of PCa [17]. Interestingly, PSMA expression also involves the neovasculature of several tumor entities, thus envisaging that the targeting of such antigen could lead to an additional antiangiogenic effect not only in PCa but also in other cancer histotypes. Therefore, PSMA appears a very promising target and indeed is currently exploited for both imaging and therapeutic purposes [18]. It has been demonstrated that NK-92/CAR cells exhibit appreciable anti-tumor activity in several clinical trials [13] against different hematological and solid tumors. However, the use of NK-92/CAR cells in treating PCa has not yet been investigated. In this novel approach, using a PSMA-specific CAR [19] to redirect NK-92 cells, we showed that genetically modified NK-92/CAR cells acquired the ability to specifically and effectively lyse PSMA-expressing PCa cells, in contrast to parental NK-92 cells. Furthermore, adoptive transfer of gene-modified NK-92 cells reduced tumor growth in different PCa mouse tumor models, and significantly enhanced survival.

In conclusion, our data pave the way to a new therapeutic perspective for PCa based on the development of an off-the-shelf, renewable product that can be advanced as a reliable, efficient and relatively low cost “universal” platform for adoptive cell therapy.

## 2. Materials and Methods

### 2.1. Cell Lines and Culture Conditions

LNCaP and PC3 (both human prostate carcinoma cell lines), 293T (human embryonic kidney cell line), K562 (human myelogenous leukemia cell line), and NK-92 (human malignant non-Hodgkin’s lymphoma natural killer cell line) were obtained from the American Type Culture Collection (ATCC). PC3-PSMA, a PC3 derivative cell line stably expressing human PSMA, has been previously described [20]. LNCaP, PC3, PC3-PSMA, and K562 were cultured in RPMI 1640 medium, while DMEM medium was used for 293T; all cell lines were enriched with 10% heat-inactivated fetal bovine serum (FBS, Gibco). Firefly luciferase (fluc)-expressing PC3 and PC3-PSMA cell derivatives were obtained by viral transduction as previously described [19]. NK-92 cells were grown in alpha MEM medium supplemented with 12.5% horse serum and 12.5% FBS, and 200 IU/mL hIL-2 (Proleukin; Novartis Pharma). All media were supplemented with 2 mM L-Glutamine, 100 U/mL penicillin, and 100 μg/mL streptomycin (all from Lonza), and maintained at 37 °C in a humidified 5% CO_2_ incubator.

### 2.2. Generation of CAR-Expressing NK-92 Cells

The anti-PSMA CAR/eGFP lentiviral transfer vector (LV), and viral particle production in 293 T cells have been previously described [19]. NK-92 cells were transduced with viral particles for 18 h at 37 °C and 5% CO_2_ in the presence of protamine sulfate (40 ug/mL; Sigma-Aldrich) and hIL-2 (500 IU/mL). Thereafter, the cells were maintained in culture by supplying fresh complete medium containing hIL-2 (200 IU/mL). Enrichment of CAR-expressing NK-92 cells was carried out by flow cytometry cell sorting with Moflo Astrios (Beckman Coulter) based on eGFP expression intensity, and the resulting population was referred to as NK-92/CAR cells.

### 2.3. Irradiation of NK-92 Cells

NK-92/CAR and parental NK-92 cells were collected by centrifugation, counted, washed, resuspended in fresh growth medium, and irradiated with 5 or 10 Gy using a γ-ray irradiator (Schering). For in vitro proliferation and cytotoxicity assays, cells were irradiated with 10 Gy, washed, resuspended in fresh growth medium, and cultured for up to 8 days. Proliferation was analyzed by counting viable cells at different time points using trypan blue exclusion. For in vivo experiments, cells were irradiated with 10 Gy and used directly.

### 2.4. Antibodies

CAR-expressing cells were labeled with the anti-c-myc mAb (clone 9E10; Sigma-Aldrich) or the isotype control (mouse IgG1; Southern Biotech), followed by a secondary antibody (PE-conjugated goat anti-mouse IgG; Southern Biotech). Cell surface markers were labeled using PE-conjugated antibodies to CD85j, NKG2D, CD158d, CD27, and with the relative isotype controls (BioLegend). Flow cytometry analyses were conducted using a FACSCalibur flow cytometer and FlowJO software (BD Biosciences).

### 2.5. Cytokine Release Assay

IFN-γ production was assessed by an ELISA IFN-γ Screening Set (Thermo Scientific), according to the manufacturer’s instructions. Briefly, 1 × 10^5^ NK-92/CAR cells were seeded in triplicate together with 1 × 10^5^ target cells (PC3 or PC3-PSMA), in 96-well round bottom plates. Cytokine secretion was measured after 12 h of incubation. Negative and positive controls were represented by NK-92/CAR cells that remained unstimulated or were treated with 40 ng/mL of PMA and 4 mg/mL of ionomycin (Sigma-Aldrich), respectively. Parental NK-92 was included for comparison. Supernatants were then analyzed using a VICTOR X4 (PerkinElmer).

### 2.6. Degranulation Assay

Degranulation of NK-92/CAR and parental NK-92 cells was induced upon interaction with target cells at 1:1 ratio for 5 h at 37 °C, and was assessed by measuring the surface expression of CD107a with an APC-labelled anti-CD107a detection antibody (Biolegend). Effector cells stimulated with PMA/ionomycin or kept without targets served as controls.

### 2.7. Cytotoxicity Assay

The cytotoxic activity of NK-92/CAR and parental NK-92 cells was assessed in a standard 4h ^51^Cr-release assay. In brief, effector cells were incubated with 2 × 10^3 51^Cr-labeled PC3-PSMA, PC3, LNCaP, and K562 cells at various E/T ratios in triplicate wells of 96-well round-bottom plates, with or without a NKG2D blocking antibody (clone 1D11; Biolegend). Antibody was added to the effector cells at a concentration of 50 μg/mL, and incubated for 1 h at 37 °C prior to the addition of the targets. The cytotoxic activity of NK-92/CAR and parental NK-92 cells was evaluated before and after irradiation. The percentage of specific ^51^Cr release into the supernatant was assessed as previously described [21].

### 2.8. In Vivo Studies

All in vivo experiments involved 6- to 8-week-old NOD/SCID common γ chain knockout (NSG; Charles River) male mice, which were housed in our Specific Pathogen Free (SPF) animal facility. Procedures involving animals and their care were in conformity with institutional guidelines that comply with national and international laws and policies (D.L. 26/2014 and subsequent implementing circulars), and the experimental protocol (Authorization n. 1143/2015-PR) was approved by the Italian Ministry of Health. During in vivo experiments, animals in all experimental groups were examined daily for a decrease in physical activity and other signs of disease; severely ill animals (weight loss exceeding 15%, lethargy, ruffled hair, low temperature) were euthanized by carbon dioxide overdose. 

*Winn assay.* Winn assay was performed by injecting mice subcutaneously (s.c.) with 5 × 10^6^ PC3 or PC3-PSMA cells, mixed with either RPMI, NK-92/CAR or NK-92 cells (5 × 10^6^/mouse; 6 mice/group). Tumor volume was calculated according to the following equation: V (mm^3^) = (d^2^ * D)/2, where d (mm) and D (mm) are the smallest and largest perpendicular tumor diameters, respectively, as assessed by caliper measurement.

*Systemic treatment of subcutaneous prostate tumors.* To assess the therapeutic activity of systemically administered NK-92/CAR cells in a subcutaneous prostate tumor model, mice were injected s.c. with 5 × 10^6^ PC3-PSMA cells and 4 days later started intravenous (i.v.) treatment with effector cells (10 × 10^6^/mouse; 6 mice/group); cell administration was repeated for 3 times at alternate days over a one week interval. Specificity of NK-92/CAR cells was assessed in mice injected s.c. with 5 × 10^6^ PC3 cells, while tumor-bearing mice left untreated or receiving parental NK-92 served as further control groups. 

*Systemic treatment of orthotopic prostate tumors.* The therapeutic impact of adoptively transferred NK-92/CAR cells was also evaluated in an orthotopic prostate tumor model. Mice were injected with 2.5 × 10^5^ bioluminescent PC3-PSMA or PC3 cells into the anterior prostatic lobe, and 2 days later started treatments as reported above. Tumor engraftment and response to therapy were evaluated by bioluminescence (BLI).

### 2.9. Statistics

Statistical analysis was performed by Student’s t test when only two value sets were compared. One-way ANOVA was used when the data involved three groups. Mice survival was compared using log-rank survival statistics. Histograms represent mean values ± standard deviation. In scatter-plot graphs, symbols indicate different samples or assays, and horizontal bars represent means ± standard deviation. *p* < 0.05, *p* < 0.01 or *p* < 0.001 were considered statistically significant and indicated by *, ** or ***, respectively. Statistical analysis was performed using GraphPad Prism 7.0 software.

## 3. Results

### 3.1. PSMA-Targeted NK-92/CAR Cells Acquire Antigen-Specific Cytotoxic Activity

**Figure 1:**
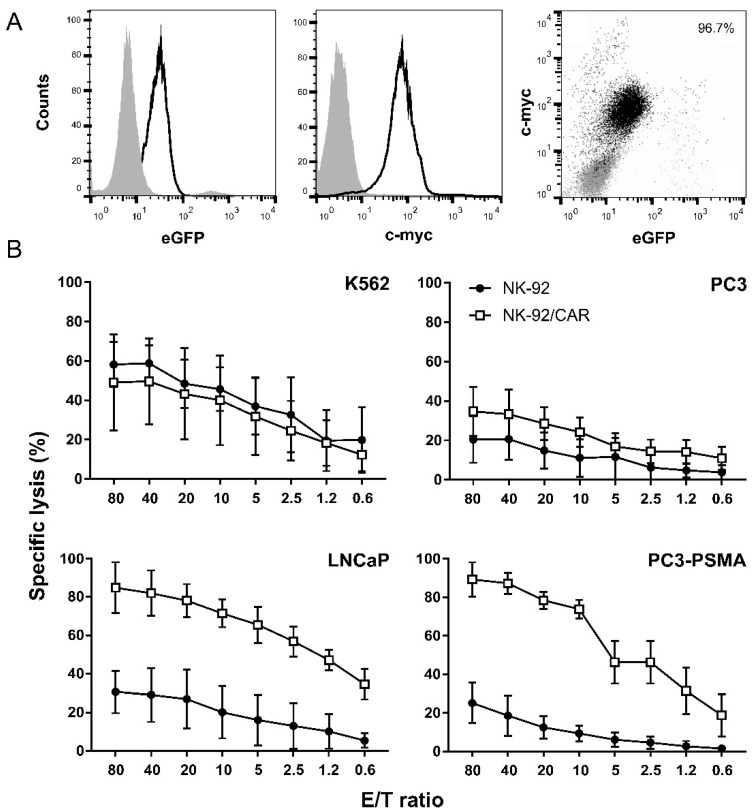
Anti-PSMA (prostate-specific membrane antigen) CAR (chimeric antigen receptors)-engineered NK-92 cells acquire high and specific cytotoxicity to antigen-expressing cancer cells. (**A**) CAR surface expression on a NK-92/CAR eGFP-based sorted and enriched population was determined by flow cytometry with an antibody to a Myc tag present in the CAR moiety [19]. Left and central panels, eGFP and c-myc expression in the sorted population, respectively (open areas); parental NK-92 cells for eGFP expression and an isotype antibody for c-myc analysis served as controls (shaded areas). Right dot plot reports the events gated on total viable cells. (**B**) Cytotoxicity by NK-92/CAR (open squares) was investigated in ^51^Cr-release assays using as targets PSMA-negative K562 and PC3 cells (upper panels), and PSMA-expressing LNCaP and PC3 (bottom panels) cells. Parental NK-92 cells were included for comparison (full circles). Results are reported as mean values ± SD (n = 3) at different effector to target (E/T) ratios.

To express the anti-PSMA CAR, we used an LV carrying a bidirectional promoter that drives the simultaneous expression of the CAR molecule, and the eGFP reporter gene (17). After generation of lentiviral particles and transduction of NK-92 cells, the eGFP-expressing NK-92/CAR subset underwent enrichment by flow cytometry sorting, leading to a virtually 100% CAR-positive cell population (Figure 1A). As NK-92 cells are endowed with intrinsic killing activity against the NK-sensitive K562 cell line, we initially compared the natural cytotoxicity of the parental and the transduced populations. Both NK-92 and NK-92/CAR cells disclosed a relevant and overlapping lysis against K562 cells (Figure 1B), thus demonstrating that the transduction and selection procedures do not impinge on the intrinsic properties of NK-92 cells. Next, we evaluated the lytic activity of the retargeted NK-92/CAR cells towards different prostate tumor targets. NK-92/CAR cells showed, even at low E/T ratios, an extremely high cytotoxicity to PC3 cells stably transfected and expressing PSMA at high intensity, which instead turned out resistant to parental NK-92 cells (Figure 1B). Likewise and more importantly, LNCaP cells, which naturally harbor the PSMA antigen, were selectively killed by NK-92/CAR cells but not the parental NK-92 counterparts (Figure 1B). As further proof of specificity, both NK-92/CAR and NK-92 cells failed to lyse PSMA-negative PC3 cells included as a control (Figure 1B). Overall, data indicate that the PSMA-specific CAR is fully functional within NK-92 cells and confers antigen-selective redirected and enhanced activity.

### 3.2. Irradiation Is a Safe Measure to Prevent NK-92/CAR Cell Proliferation

**Figure 2:**
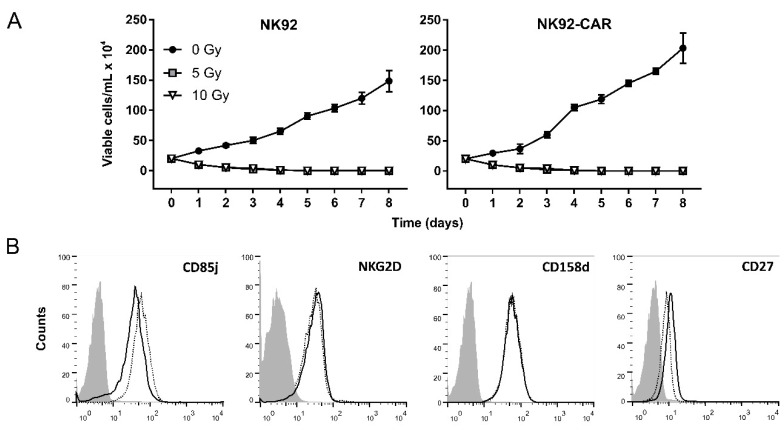
The irradiation of CAR-transduced or parental NK-92 cells fully blocks the proliferation but not impinges on phenotype. (**A**) Viability of NK-92 (left panel) and NK-92/CAR (right panel) cells undergoing or not γ-irradiation at 5 or 10 Gy. Proliferation was analyzed by counting viable cells at the indicated time points using trypan blue exclusion. Results are reported as mean values ± SD of 3 independent experiments. (**B**) Surface expression of different NK (natural killer) cells’ activation/inhibition receptors (CD85j, NKG2D, CD158d, and CD27) after γ-irradiation was analyzed by flow cytometry staining with appropriate antibodies. Solid lines refer to NK-92/CAR cells, while dotted lines represent parental counterparts.

In phase I clinical trials with untargeted NK-92 cells, a 10 Gy irradiation step of the effector population prior to infusion had been included as a safety measure to prevent permanent engraftment [9,11]. It is quite conceivable that similar safety measures are requested for the clinical application of retargeted NK-92 cells. Hence, we decided to test the effects of γ-irradiation on growth and phenotype of NK-92/CAR cells. After exposure to either 5 or 10 Gy γ-irradiation, further replication was prevented and the number of viable NK-92/CAR cells gradually declined, with living cells no longer detectable after 5–7 days (Figure 2A). Then, to investigate the effects of irradiation on the NK-92/CAR cell phenotype, we evaluated the potential modulation of some activation and inhibition receptors typical of NK-92 cells [22]. No difference in the expression of CD27, NKG2D, CD158d or CD85 cell markers was noted between NK-92/CAR and parental NK-92 cells (Figure 2B), thus demonstrating that γ-irradiation does not alter the phenotype of NK-92 cells.

### 3.3. Irradiated NK-92/CAR Cells Undergo Degranulation and IFN-γ Release Upon Interaction with PSMA-Expressing Targets

**Figure 3:**
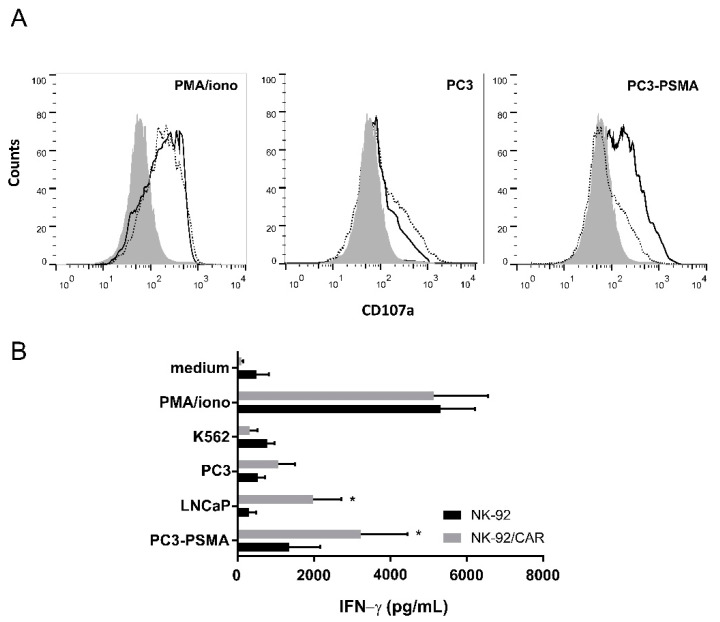
Degranulation and IFN-γ secretion by irradiated NK-92/CAR cells upon engagement with PSMA-expressing prostate cancer cells. (**A**) Degranulation of NK-92/CAR cells (solid lines) was analyzed by flow cytometry assessment of CD107a surface expression after 5h of co-culture with PC3 or PC3-PSMA cells. Parental NK-92 cells (dotted lines) were included for comparison. Effector cells kept alone (shaded areas) or stimulated with PMA/ionomycin served as basal and positive controls, respectively. (**B**) IFN-γ release was analyzed in supernatants of NK-92/CAR cells stimulated with PSMA-negative or -positive cancer cell lines. NK-92/CAR cells treated with PMA/ionomycin served as positive controls. Parental NK-92 cells were included for comparison. Results are reported as mean values ± SD of 3 independent experiments. * *p* < 0.05.

NK and cytotoxic T cell-mediated lysis upon recognition of target cells requires efficient triggering of the effector cells, which ultimately culminates in the exocytosis of lytic granules. To investigate the degranulation of irradiated NK-92/CAR and parental NK-92 cells upon interaction with target cells, we measured the surface expression of the lysosomal-associated membrane protein LAMP-1 (CD107a). While a non-specific stimulus represented by the combination of PMA and ionomycin triggered a massive and fully overlapping degranulation in either populations, thus further stressing the concept that neither CAR transduction nor irradiation impinge on NK-92 functionality, PC3 cells produced only marginal effects in line with the low recognition of such targets that lack PSMA expression. Conversely, when the PSMA-expressing PC3 variant was used, NK-92/CAR cells underwent a strong upregulation of CD107a expression that was however absent in parental NK-92 cells (Figure 3A). In parallel, we also tested IFN-γ release by NK-92/CAR and NK-92 cells following target engagement. Results of this assay fully mirrored those already obtained with CD107a expression analysis, as PMA/ionomycin massively but non-specifically stimulated either effector populations, PC3 cells were negligibly recognized, while only NK-92/CAR cells produced significant levels of IFN-γ upon interaction with PSMA+ targets (Figure 3B). Overall, these data demonstrate that the anti-PSMA CAR is selectively activated by the specific antigen, and that the resulting signaling chain correctly proceeds from the membrane up to the nucleus even in irradiated cells.

### 3.4. NK-92/CAR Cells Retain a High and Specific Target Killing Activity upon Irradiation

**Figure 4:**
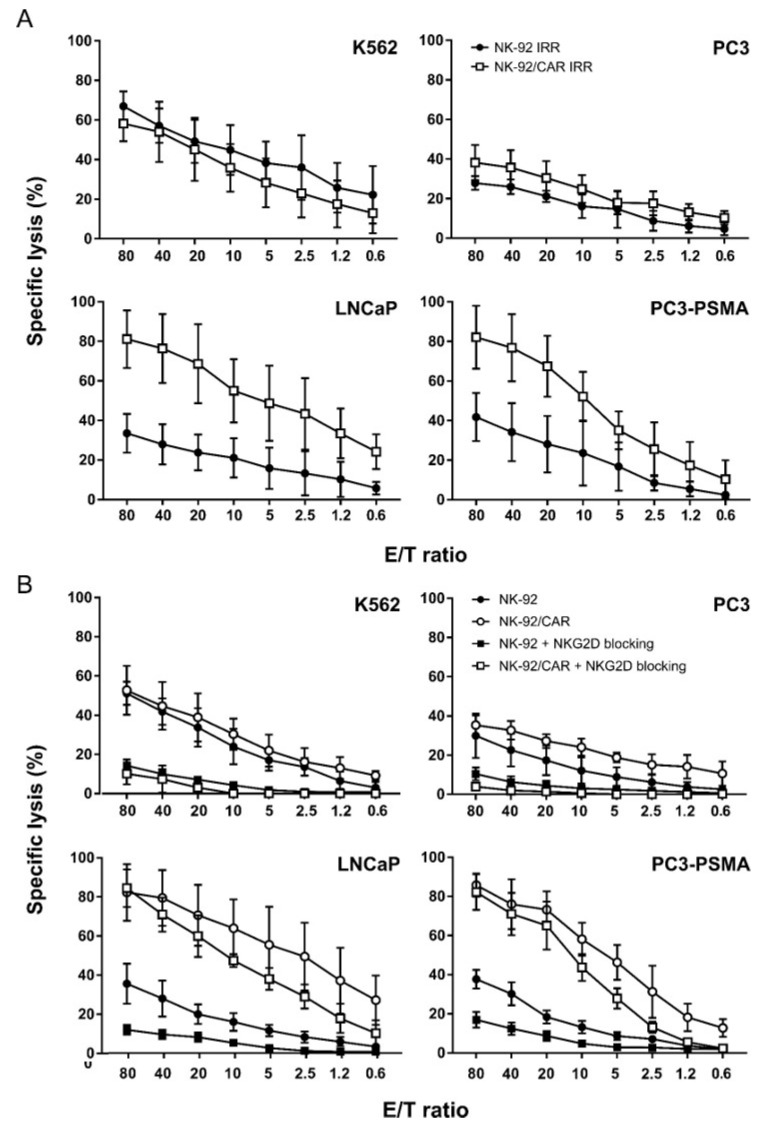
Lytic activity of irradiated NK-92/CAR cells before and after NKG2D blocking. (**A**) Cytotoxicity of 10 Gy-irradiated NK-92/CAR (open squares) and NK-92 (filled circles) cells was investigated against K562, PC3, LNCaP, and PC3-PSMA cells, at different E/T ratios. (**B**) To evaluate the effects of NKG2D blocking, irradiated NK-92/CAR (open squares) and parental NK-92 (filled squares) cells were pre-treated with anti-NKG2D antibody for 1 h before incubation with K562, PC3, LNCaP, and PC3-PSMA targets. Irradiated non-blocked CAR-transduced (open circles) and wild-type (filled circles) effectors were included for comparison. Lysis data are shown as mean values ± SD of 3 independent experiments.

To assess the effects of irradiation on cytotoxic activity, NK-92/CAR cells were irradiated at 10 Gy and then co-incubated for 4 h with target cells. Similarly treated NK-92 parental cells were used as a control. In spite of irradiation, NK-92/CAR cells disclosed a higher cytotoxicity than NK-92 counterparts towards PSMA-expressing PC3 (*p* = 0.0012) and LnCaP (*p* < 0.0001) targets (Figure 4A). Again, this increased lytic activity was strictly antigen-specific, as NK-92/CAR and parental NK-92 cells mediated low and comparable killing of PSMA-negative PC3 targets, while irradiation did not interfere with the intrinsic NK-like activity of either effectors since K562 targets were lysed at comparable levels.

### 3.5. CAR-Mediated Cytotoxicity is Independent of NKG2D Activity in NK-92/CAR Cells

NKG2D is considered the main receptor involved in NK-92 lytic activity. To further confirm that the increased killing of NK-92/CAR cells was strictly PSMA antigen-specific and could be ascribed only to CAR activation, we analyzed the impact of NKG2D inhibition on cytotoxicity of irradiated effectors (Figure 4B). Upon NKG2D blocking, NK-92/CAR cells continued to show an elevated cytotoxic activity against PSMA-expressing targets, and the percentages of lysis remained essentially similar to those detected in the absence of blocking. On the other hand, NKG2D inhibition almost completely shut off the intrinsic lytic activity of both NK-92/CAR and NK-92 parental cells, thus suggesting that CAR- and NKG2D-mediated pathways act independently and do not interfere with each other. 

### 3.6. NK-92/CAR Cells Control the Growth of Locally Implanted Prostate Carcinoma

**Figure 5:**
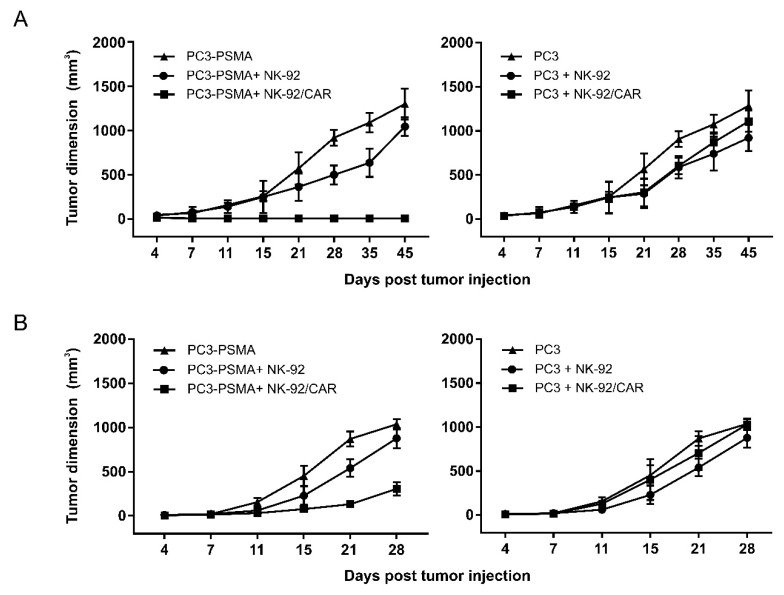
Assessment of NK-92/CAR cells’ in vivo therapeutic efficacy against locally implanted prostate cancers. (**A**) Winn assay. PC3-PSMA (left panel) and PC3 (right panel) cancer cells were inoculated s.c. in NSG (NOD/SCID common γ chain knockout) mice alone (filled triangles), or mixed with NK-92/CAR (filled squares) or parental NK-92 (filled circles) cells. Tumor growth was monitored over time by caliper measurement (6 mice/group). (**B**) Therapeutic activity against established tumors. NK-92/CAR cells or untransduced counterparts were administered i.v. in NSG mice for 3 times at alternate days, starting 4 days after s.c. injection of PC3-PSMA (left panel) or PC3 (right panel) tumor cells. Untreated animals served as control group. Symbols and groups as in a. Data are reported as mean values ± SD of tumor volumes at different time points.

The in vivo therapeutic efficacy of irradiated NK-92/CAR cells was initially evaluated using a Winn assay (Figure 5A). When NK-92/CAR effectors were co-injected s.c. with PC3-PSMA cells, tumor growth was completely blocked and no neoplastic mass developed. Conversely, co-administration of parental NK-92 cells led to only a slight but not significant delay of the tumor growth, which ultimately reached values observed in untreated animals (Figure 5A, left panel). Notably, transfer of either retargeted or parental NK-92 cells did not significantly impact on the progression of PSMA-negative PC3 tumors (Figure 5A, right panel), thus reinforcing the concept that the effects observed against antigen-expressing prostate cancer were strictly CAR-dependent. Since the Winn assay does not recapitulate a real therapeutic setting, we moved to assess the antineoplastic activity of NK-92/CAR cells against established tumors (Figure 5B). To this end, NSG mice were injected s.c. with PC3-PSMA cells and, when tumors became palpable, received systemic injections of NK-92/CAR or parental NK-92 irradiated effectors. While progressive tumor growth was observed in both untreated control mice and those receiving parental NK-92 cells, injections of NK-92/CAR effectors strongly affected prostate carcinoma development, an effect that involved all treated animals (Figure 5B, left panel). Furthermore, in this experimental setting, neither the treatment with NK-92/CAR cells nor the parental counterparts restrained the growth of PC3 tumors that lack PSMA (Figure 5B, right panel). Overall, data indicate that irradiated NK-92/CAR cells are capable of penetrating tissues and reaching distant tumor sites, where they exert antigen-specific therapeutic activities.

### 3.7. The Adoptive Transfer of NK-92/CAR Cells Is Therapeutic in an Orthotopic and Metastatic Prostate Tumor Model

**Figure 6:**
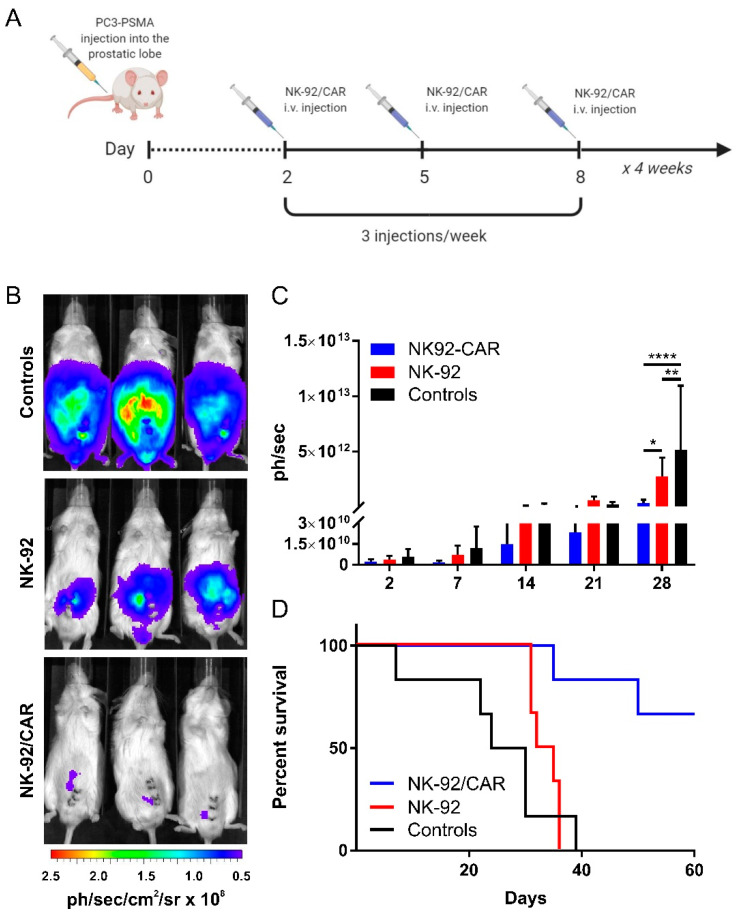
Antitumor activity of NK-92/CAR cells against orthotopic PC3-PSMA xenografts. (**A**) Representative schedule of the experiment. Luciferase-expressing PC3-PSMA cells were injected into the anterior prostatic lobe of NSG mice. Starting from two days later, mice received i.v. injections of NK-92/CAR or parental NK-92 cells thrice per week for 4 weeks. Control mice were treated with PBS, and tumor growth was monitored by BLI. (**B**) Representative images of mice from experimental groups (one mouse/group) at day 28. (**C**) Graph reports cumulative results of the regions of interest (ROI) in total body. Tumor growth was monitored as total flux (ph/sec). Graphs show mean ± SD of three independent experiments. (**D**) Cumulative Kaplan–Meier survival curves of NSG mice (untreated mice, black line; NK-92 treated mice, red line; NK-92/CAR treated mice, blue line). * *p* < 0.05, ** *p* < 0.01 and *** *p* < 0.001.

Heterotopic tumor models may always raise concerns about the real translation to clinics. Therefore, we decided to assess the therapeutic potential of irradiated and adoptively transferred NK-92/CAR cells in an orthotopic prostate tumor model that would better resemble the clinical setting. Bioluminescent PC3-PSMA prostate tumor cells were implanted into the anterior prostatic lobe of male NSG mice, followed by i.v. injections of parental NK-92 or retargeted NK-92/CAR-irradiated effectors; treatments were carried out at days 2, 5, and 8 after tumor implant, and then thrice per week for a month (Figure 6A). In control mice that received PBS only, prostate cancer developed progressively, leading in about a month to massive peritoneal metastasis. The administration of unmodified NK-92 cells was followed by a modest but significant tumor growth reduction, while only NK-92/CAR cells dramatically halted carcinoma progression as demonstrated by the significant reduction of emitted photons at day 28 (NK-92/CAR vs. NK-92, *p* = 0.005; NK-92/CAR vs. Controls, *p* < 0.0001; NK-92 vs. Controls *p* = 0.0142) (Figure 6B,C). More importantly, the transfer of NK-92/CAR cells significantly improved survival of treated mice in comparison to untreated animals (median survival 27 days, *p* = 0.0018) or animals receiving parental NK-92 (median survival 33.5 days, *p* = 0.0048) (Figure 6D), with 66% of mice still alive at the endpoint of the experiment. On the whole, these data demonstrate that NK-92/CAR cells are endowed with high and specific antitumor activity against PSMA-expressing prostate cancer cells, and that their viability and functionality is transiently preserved after γ-irradiation at a dose that prevents further effector cell replication but still permits target cell recognition and therapeutic activity in vivo.

## 4. Discussion

Undoubtedly, cancer immunotherapy is in an exciting period due to the remarkable successes achieved with the introduction of checkpoint inhibitors [23,24,25,26,27] and CAR-T cells, at least in patients with lymphoid malignancies [1,2,3]. The latter, in particular, strongly underline the concept that immune cells can be converted into powerful therapeutic agents. Nonetheless, CAR-T cell treatment is associated with heavy adverse events in a significant number of patients and, additionally, the logistics and costs of such an approach pose significant challenges for making it available to a large number of potentially suitable recipients. 

NK cells may represent safer effectors than T cells for cancer therapy. However, even in this case there are biological, logistical, and financial constrains for the application of blood NK cells as a treatment modality for cancer patients. Autologous NK cells are typically not very effective, as they are functionally silenced when encountering self-MHC antigens, and can be also frequently compromised by the underlying disease and its treatment. Indeed, NK cell function can be impaired in patients with malignant disorders, resulting in a reduced proliferative response and cytotoxic activity [28]. 

In this context, clinically applicable human cell lines, such as NK-92, may provide a valuable alternative. NK-92 cells proliferate and expand easily in culture with a doubling time of 2-4 days, and are the only cell line that is consistently and highly cytotoxic to cancer targets. This cell line has undergone an extensive preclinical assessment [29,30,31,32] and has been tested in clinical trials [9,11] that overall demonstrated the lack of infusion-associated toxicities in cancer patients. Due to the safety of infusion and feasibility of large-scale expansion, NK-92 cells are considered a promising cellular tool for ACT. Furthermore, genetic modification of NK-92 cells with CARs may lead to targeted cancer therapies, which are expected to be more specific and efficient. This latter concept has found a preclinical validation in different experimental cancer models. Indeed, NK-92 engineering with CARs to CD19/CD20 or disialoganglioside GD2, HER2 receptor tyrosine kinase, and epidermal growth factor receptor EGFR, disclosed higher activity against hematological malignancies [31,33,34] or solid tumors [7,35,36,37]. Nonetheless, the present study is the first to investigate the use of CAR-modified NK-92 cells against PCa; in particular, we aimed at modifying NK-92 cells to express a PSMA-specific CAR, and thus to convert them into a prostate cancer-specific therapeutic tool characterized by improved efficacy. In the case of the castration-resistant PCa and in its metastatic form, actually no curative interventions are available. Therefore, the main goal is to tackle the growth of the castration-resistant PCa and the metastatic disease. 

In fact, we showed that CAR engineering endows NK-92 cells with PSMA-specific recognition capacity, and strongly enhances their lytic activity against relevant targets without interfering with the inherent NK-like activity. Since such characteristics are maintained after irradiation, this may be relevant for future clinical application of NK-92/CAR cells, where irradiation may be included as a safety measure as reported in previous phase I clinical trials with untargeted NK-92 counterparts [8,9,10,11]. Although information about the clinical application of irradiated NK-92/CAR cells is still scarce [38], such a concept seems supported by data from a recent clinical trial in relapsed and refractory acute myeloid leukemia [39] and in several other early phase clinical trials with CAR-engineered NK-92 cells that are presently ongoing in Europe, China, and the US. Regarding glioblastoma (GB), where patients’ median survival remains less than 1 year, despite multimodal regimes, a phase I clinical trial “Intracranial Injection of NK-92/5.28.z Cells in Patients With Recurrent HER2-positive Glioblastoma” (i.e., CAR2BRAIN, NCT03383978, clinicaltriaols.gov) was started in 2017, in order to evaluate the feasibility, safety and tolerability of the direct injection of irradiated NK-92 CAR-recognizing ErbB2 antigen into the wall of the resection cavity [40]. In this case the local therapy approach was chosen to increase the accumulation of the engineered cells at the site of the lesion overcoming the problems associated with the blood–brain barrier passage.

Two additional aspects are noteworthy: first, the full functionality of the CAR not only confers antigen-specific and increased lytic activity but also leads to significant IFN-γ secretion. This is typical for activated NK cells, and could in principle result in recruitment and maturation of antigen-presenting cells, and enhancement of endogenous antitumor immune responses in an immunocompetent host [41]. Second, NK-92 cells can spontaneously kill tumors by recognizing diverse ligands via a variety of NK activating receptors. Among these, the NKG2D receptor is one of the major signaling pathways involved in cancer immunosurveillance [41,42,43,44]. As we demonstrated that CAR- and NKG2D-dependent activation pathways do not reciprocally interfere, it is conceivable that they can act in concert in engineered NK-92 cells to potentiate tumor destruction. 

A final point deserves attention. Upon in vivo transfer, irradiated NK-92/CAR cells retain target cell specificity and therapeutic activity. Currently, irradiation of the cells before in vivo application is the usual safety method employed in the clinical setting [9,11] to block proliferation and prevent permanent engraftment of NK-92 cells. While this approach has minimal effects on their short-term functionality, it negatively impacts on their in vivo persistence and long-term antitumor efficacy. Hence, repeated infusions of irradiated NK-92 cells are required as a means to circumvent their limited survival, but the approach has drawbacks in terms of costs and patient compliance. In this regard, introducing a suicide gene into NK-92/CAR cells might represent a valid safety option alternative to irradiation, because it would likely allow a single administration of effectors that are subsequently amenable to being completely switched off after exerting their therapeutic activity or in the case of severe, unwanted toxicity [42,43,44,45,46,47]. 

## 5. Conclusions

Overall, the results of this study highlight the potentialities of PSMA-specific CAR-modified NK-92 cells as a novel and exciting perspective for prostate cancer adoptive immunotherapy. Moreover, the potent antitumor activity, the immediate availability as a fully defined and characterized cell product, and the lack of obvious risks of manufacturing failures suggest that these cells can be advanced as a valid and cost-effective alternative to CAR-modified T cells. Finally, the robust ex vivo expansion of NK-92 cells to high numbers and their exquisite safety profile, as well as the ease of genetic modification, make this cell line an ideal platform for the development of off-the-shelf therapeutic CAR-engineered variants to target other solid tumors.

## Abbreviations

^51^Cr, chromium-51; ACT, adoptive cancer immunotherapy; ATCC, American Type Culture Collection; Alpha MEM, minimum essential medium eagle, alpha modification; BLI, bioluminescence; CAR-T, chimeric antigen receptor T; DMEM, Dulbecco’s Modified Eagle Medium; E/T, Effector/Target; eGFP, enhanced green gluorescent protein; EGFR, epidermal growth factor Receptor; FBS, Fetal Bovine Serum; Fluc, Firefly luciferase; GD2, disialoganglioside; GMP, Good Manufacturing Practice; HER2, human epidermal growth factor 2; i.v., intravenous; IFN-γ, interferon-γ; LAMP-1, lysosomal associated membrane protein; LV, lentiviral transfer vector; mAb, monoclonal antibody; MHC, major histocompatibility complex; NK, natural killer; NKG2D, natural-killer group 2 member D; NSG, NOD/SCID common γ chain knockout; PBS, phosphate buffered saline; PCa, prostate cancer; PMA, phorbol-myristate-acetate; PSMA, human prostate-specific membrane antigen; ROI, regions of interest; RPMI, Roswell Park Memorial Institute; s.c., subcutaneously; SD, standard deviation; SPF, specific pathogen free.
